# A Comprehensive Review of Interstitial Lung Abnormalities

**DOI:** 10.1111/resp.70026

**Published:** 2025-03-16

**Authors:** Yuben Moodley, John A. Mackintosh

**Affiliations:** ^1^ University of Western Australia Perth Western Australia Australia; ^2^ Department of Respiratory Medicine Fiona Stanley Hospital Murdoch Western Australia Australia; ^3^ Institute for Respiratory Health Nedlands Western Australia Australia; ^4^ Centre for Research Excellence in Pulmonary Fibrosis Camperdown New South Wales Australia; ^5^ Department of Thoracic Medicine The Prince Charles Hospital Brisbane Queensland Australia; ^6^ Faculty of Medicine University of Queensland Brisbane Queensland Australia

**Keywords:** interstitial lung abnormalities, pulmonary fibrosis, radiology

## Abstract

Interstitial lung abnormalities (ILAs) represent radiological entities that comprise changes compatible with an interstitial process, occurring in individuals not suspected to have interstitial lung disease (ILD). The prevalence of ILAs ranges from 2.5% to 16.7% in lung cancer screening and population‐based cohorts. ILAs have consistently been associated with mortality. Risk factors include older age, smoking, and genetic polymorphisms such as MUC5B. Progression of ILAs occurs in 20%–76% of cases over 2–6 years of follow‐up. The clinical approach to ILAs involves risk stratification based on radiological features, extent of involvement, and associated clinical and physiological findings. ILAs pose a significant challenge in distinguishing inconsequential radiological findings from early ILD. This review summarises the current understanding of ILAs, including prevalence, risk factors, progression, associated biomarkers, and clinical management strategies.

## 1 Introduction

Recognition of interstitial lung abnormalities (ILAs) has increased with the expanding use of computed tomography (CT) in lung cancer screening and other clinical settings. Defined as incidental radiological findings that may be suggestive of an interstitial process occurring in individuals not known or suspected to have interstitial lung disease (ILD), the importance of ILAs lies in their potential to identify individuals at risk of developing clinically significant ILD, particularly idiopathic pulmonary fibrosis (IPF), which might enable early disease‐modifying therapy prior to the development of clinically recognisable disease.

This review aims to provide a comprehensive overview of ILAs, synthesising current knowledge on their prevalence, risk factors, progression, associated biomarkers, and clinical implications. We will explore the challenges in differentiating inconsequential radiological findings from early ILD and discuss current approaches to risk stratification and management. By examining the latest evidence, we seek to inform clinicians and researchers about the significance of ILAs to guide clinical approach, in preparation for wider implementation of lung cancer screening.

## 2 What Is and Is Not an ILA?

### 2.1 What Is an ILA?

Interstitial lung abnormalities (ILAs) are a radiological entity comprising changes compatible with an interstitial process, occurring in a person not suspected to have an interstitial lung disease (ILD). This usually occurs in the absence of symptoms and impairment in lung function. The conundrum posed by an ILA is whether the change represents an early ILD at risk of progression or clinical consequence, or simply a radiological abnormality.

**FIGURE 1:**
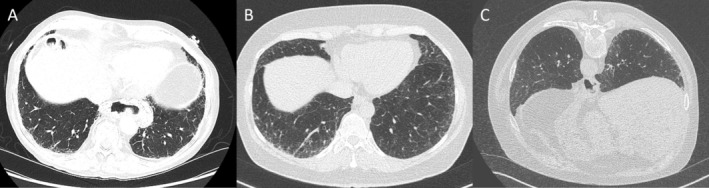
Interstitial lung abnormality (ILA) examples. (A) Subpleural, basal predominant fibrotic ILA discovered in an 88‐year‐old male undergoing evaluation for transcatheter aortic valve implantation. (B) Subpleural, basal predominant, ground glass ILA discovered in a 58‐year‐old female through CT coronary artery calcium score evaluation. (C) Subpleural, basal predominant, mixed ground‐glass and fibrotic ILA discovered in a 75‐year‐old male through CT colonography.

**FIGURE 2:**
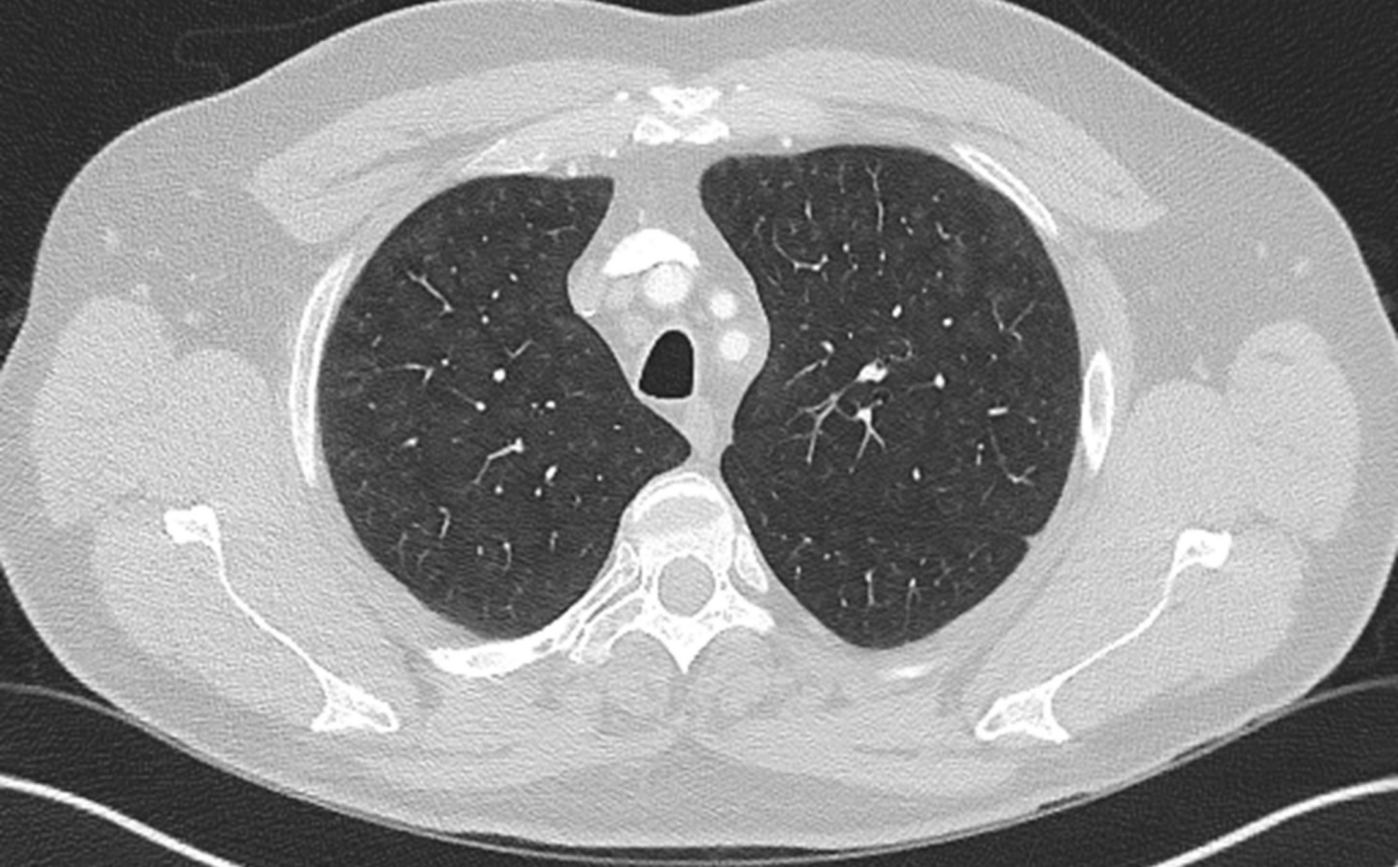
Respiratory bronchiolitis interstitial lung disease in an active cigarette smoker.

**FIGURE 3:**
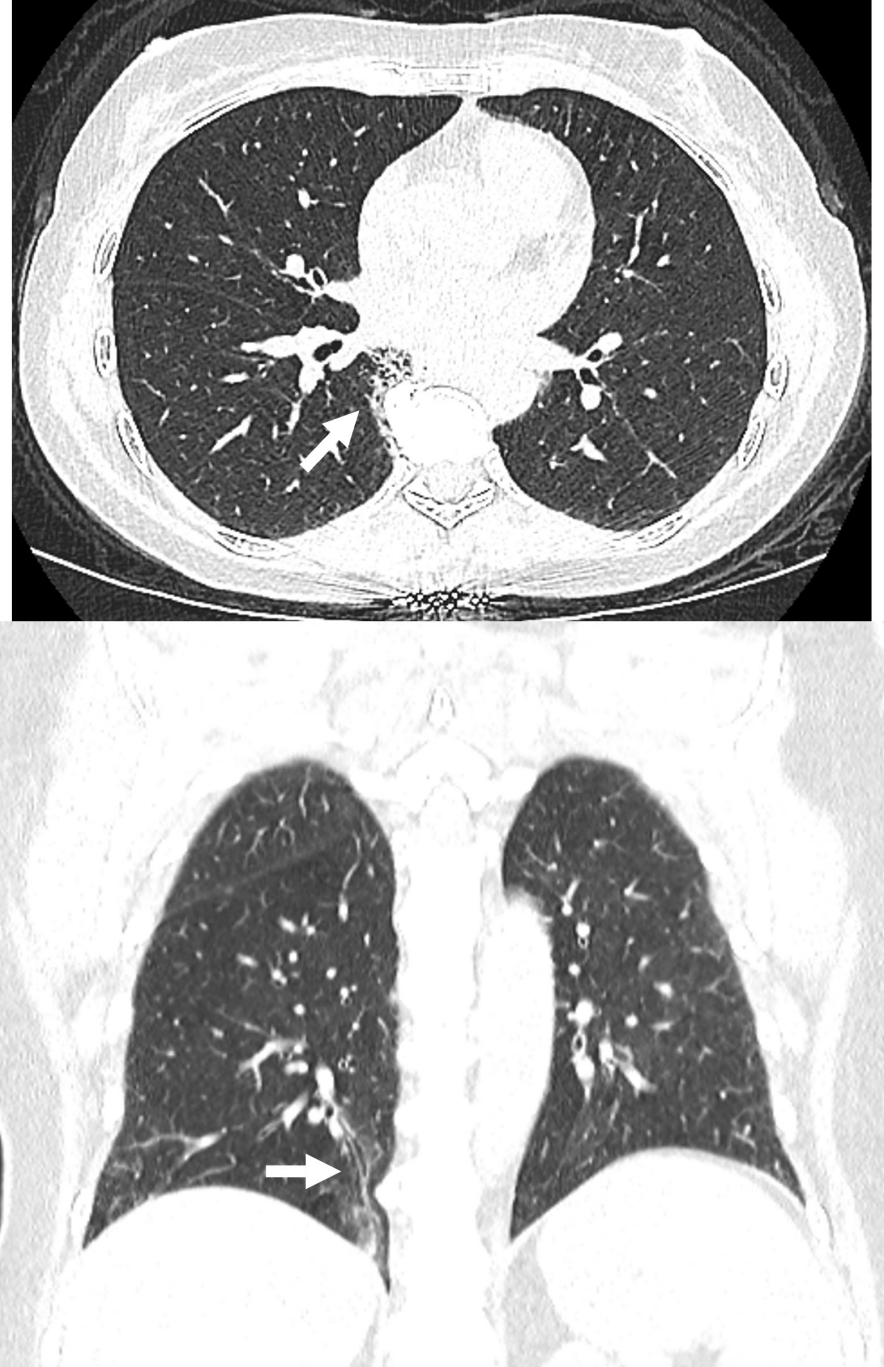
Vertebral osteophyte associated pulmonary fibrosis.

In July 2020, after numerous reports of ILAs in cross‐sectional and lung cancer screening studies, the Fleischner Society published a Position Paper outlining the criteria to define an ILA (Table 1) [1]. Three criteria were proposed: (1) the incidental discovery of essentially any interstitial radiological abnormality of a non‐dependent nature, (2) involving at least 5% of a lung zone, (3) in an individual not suspected to have an ILD (Figure 1). Certain radiological abnormalities and conditions were excluded from this definition. ILAs are largely considered in reference to ILD, and so atelectasis, focal abnormalities, interstitial oedema, and infective changes are excluded from the ILA entity. Diffuse centrilobular ground‐glass nodularity in a smoker, representative of respiratory bronchiolitis ILD (RBILD), is a defined clinical entity differentiating it from an ILA (Figure 2). Fibrosis adjacent to vertebral osteophytes is considered a benign entity, resulting from a mechanical insult to the lung (Figure 3). Vertebral osteophytes are largely an aging phenomenon, with adjacent non‐progressive pulmonary fibrosis occurring in 41% of cases in one series [2]. While osteophyte‐induced fibrosis can be dismissed, it is necessary to ensure that interstitial changes distant to the osteophyte are not ignored. Additionally, honeycombing is seldom present in osteophyte‐induced fibrosis [2], and therefore is a finding requiring more careful consideration.

**TABLE 1:** Fleischner society criteria to define an ILA.

Criteria to define an ILA (all must be satisfied)
Incidental discovery in a person not suspected to have an ILD (*does not necessitate the absence of respiratory symptoms or signs)
Any of the following radiological features: (ground glass, reticulation, lung distortion, traction bronchiectasis, honeycombing, non‐emphysematous cysts)
Non‐dependent

*Note*: Involving at least 5% of a lung zone.

In the evaluation of an ILA, it is necessary to exclude dependent changes. Lung cancer screening, which potentially represents the largest source of ILAs, typically adopts low‐dose supine scanning. In one lung cancer screening study, 46% of identified interstitial abnormalities resolved on prone high‐resolution computed tomography (HRCT) [3]. Minor interstitial changes identified on low‐dose CT should therefore be subjected to prone HRCT imaging. The authors of the Fleischner ILA Position Paper note that the 5% threshold is arbitrary and imprecise and therefore perhaps clinically impractical. The intention is to avoid clinical attention to inconsequential radiological changes.

### 2.2 ILA Histopathology

While ILAs are a radiological entity, they are not simply a CT artefact. A small number of studies have evaluated the histological correlates of radiological ILAs. Verleden et al. reviewed the histology of eight ILAs discovered in unused donor lungs and tumour resection specimens [4]. The cohort aged 60–83 years had reticulation and ground glass opacity on CT. Interestingly, 83% of histological specimens from the group had fibrosis, with fibroblastic foci identified in approximately 20%. Miller et al. retrospectively reviewed the histopathology of lung nodule resection specimens in people without a history of ILD [5]. Of 26 cases with a radiologic ILA, histopathologic fibrosis was present in 73% and fibroblastic foci in 28%. Chae et al. reviewed the histology of 45 ILAs subjected to surgical lung biopsy, after exclusion of cases not meeting the ILA definition and those with clinically significant ILD [6]. Of the 36 ILAs that were radiologically fibrotic, 25 satisfied the histological criteria for definite or probable usual interstitial pneumonia (UIP), with 8 indeterminate for UIP and 3 inconsistent with UIP. In this study, fibrotic ILAs were associated with a higher risk of progression and death. These studies confirm that ILAs are often histologically fibrotic. However, confirmation of histological fibrosis, including UIP‐type, does not imply that the ILA is indicative of idiopathic pulmonary fibrosis nor that it will be progressive. Based on the findings of background fibrosis in lung cancer resection specimens [7, 8], it is suspected that many ILAs in current/former smokers are representative of smoking‐related fibrosis/macrophage accumulation [1]. Whether histological assessment enables ILAs to be differentiated from ILD will be discussed later in this review.

## 3 ILA Prevalence

### 3.1 Lung Cancer Screening

ILAs rose to prominence largely as a consequence of their identification through lung cancer screening (Table 2). Lung cancer screening studies recruit asymptomatic individuals at heightened risk of lung cancer owing to age and current or former smoking history, which are also risk factors for ILD. Typically, a baseline scan is performed and repeated yearly or biannually, for a total of 2 years of surveillance. The prevalence of ILAs identified through lung cancer screening ranges between 4.0% and 16.7% [3, 20, 21, 22, 23, 24, 25]. Lung cancer screening will soon be implemented in Australia. It is estimated that approximately 930,500 individuals will be eligible for screening [26]. Assuming a modest prevalence of ILAs of 5% in this cohort equates to approximately 46,500 individuals discovered to have an ILA. The burden of this incidental finding through lung cancer screening on the healthcare system is likely to be significant.

**TABLE 2:** Summary of data relating to interstitial lung abnormalities in population and lung cancer screening cohorts.

	Population cohorts					Lung cancer screening cohorts				
	MESA [9, 10, 11, 12]	FHS [13, 14, 15, 16, 17, 18]	AGES‐Reykjavik [17, 18]	COPDGene [17, 18, 19]	ECLIPSE [13, 17]	Nagano [3]	NLST [20, 21]	MILD [22]	DLCST [23]	QLCSS [24]
Participants assessed for ILA	6197	2633	5320	2068	1670	3079	884	692	1990	256
Duration of CT follow‐up	12 years	6 years	5 years	5 years	Not applicable	4 years	2 years	3 years	4 years	2 years
Prevalence of ILA	4.3%–7.3%	7%	7%	8%	9%	2.5%	9.7%	7%	16.7%	7.8%
Progression of ILA	Not reported	76%	73%	Not reported	Not reported	44%	20.3%	25%	Not reported	5%
Mortality associated with ILA	HR 1.58, 95% CI, 1.39–1.79	HR 2.7, 95% CI 1.1–6.5	HR 1.3, 95% CI, 1.2–1.4	HR 1.8, 95% CI, 1.1–2.8	HR 1.4, 95% CI 1.1–2.0	Not reported	Not reported	Not reported	HR 2.0, 95% CI, 1.4–2.7	Not reported
ILA associations	Older age Ever‐smoking MUC5B promoter Telomere length Hiatus hernia MMP7 IL‐6	Older age Smoking MUC5B promoter Telomere length Monocyte count	Older age MUC5B promoter Telomere length Monocyte count	Older age Telomere length Monocyte count	Monocyte count	Male Smoking history KL‐6 SP‐A SP‐D		Older age Male Current smoking	Older age	Crackles Less emphysema

Abbreviations: AGES‐Reykjavik, Age, Gene/Environment Susceptibility; COPDGene, Chronic Obstructive Pulmonary Disease Gene Study; DLCST, Danish Lung Cancer Screening Trial; ECLIPSE, Evaluation of COPD Longitudinally to Identify Predictive Surrogate Endpoints; FHS, Framingham Heart Study; MESA, Multi‐Ethnic Study of Atherosclerosis; MILD, Multicentric Italian Lung Detection trial; NLST, National Lung Screening Trial; QLCSS, Queensland Lung Cancer Screening Trial.

### 3.2 General Population

Numerous population‐based cohort studies in which participants are subjected to CT imaging reveal a similar prevalence of ILAs to those identified through lung cancer screening (Table 2) [9, 13, 14, 15, 27, 28, 29, 30]. However, these studies by design have longer durations of follow‐up, allowing for the progression of ILAs and the development of new incident ILAs to be assessed past the conventional two‐year interval of surveillance adopted in lung cancer screening. Consequently, higher rates of ILA progression are reported in these studies, up to 63% at 5 years in the AGES‐Reykjavik cohort [13].

ILAs detected through lung cancer screening and population‐based cohort studies represent only the tip of the iceberg of the full extent of ILA prevalence. With the widespread availability of CT imaging and with ever‐increasing indications for CT scanning, ILAs may also be detected through routine thoracic as well as abdominal CT imaging. Achaih et al. reported the prevalence of early fibrotic ILAs (reticulation ± ground glass opacities, excluding traction bronchiectasis and honeycombing) on routine thoracic CTs in the Oxfordshire region [31]. Over a five‐year interval, 40,711 thoracic CTs were performed for non‐ILD indications. Early fibrotic ILAs were identified in 3.1% of those scans. Where follow‐up CT imaging was available, 43.4% of ILAs demonstrated radiological progression. The prevalence of ILA in this cohort is likely to have been underestimated, owing to the exclusion of people over the age of 75 years and a requirement that the reporting radiologist needed to have documented an interstitial abnormality at the time of reporting.

### 3.3 Asymptomatic Family Members of People With ILD

As emphasised earlier, the critical aspect of what is an ILA is its identification in an individual not known or suspected to have an ILD. While interstitial abnormalities might be identified in the asymptomatic relatives of people with ILD, it has been proposed that their heightened risk of ILD should exclude them from an ILA label. It has been suggested that such a finding be termed pre‐clinical ILD. Salisbury et al. screened asymptomatic individuals from families where more than two members had been diagnosed with pulmonary fibrosis, equating to a diagnosis of familial pulmonary fibrosis [32]. Among the asymptomatic family members, ILAs were identified at a prevalence of 23%, significantly higher than in the above‐mentioned population cohort and lung cancer screening studies. ILAs were detected at an age that was, on average, 7 years earlier than the proband's age of diagnosis. At 5 years, 63% of ILAs had progressed, with a significant number developing into a definite ILD or extensive change comprising > 5% honeycombing and/or > 30% lung involvement. There was a 6% incidence of new ILA in the cohort during follow‐up.

Mathai et al. corroborated the findings of Salisbury et al., inviting 494 people, self‐reporting as “unaffected” from 263 families to undergo HRCT imaging [33]. Interstitial changes were identified in 93 (18.8%) individuals, with 77 meeting criteria for pre‐clinical pulmonary fibrosis. Pre‐clinical pulmonary fibrosis was most prevalent in those 60 years of age and older, and in the presence of *MUC5b* risk allele carriage. Over half the cohort underwent follow‐up HRCT. During follow‐up, 16 new cases of pre‐clinical pulmonary fibrosis were identified, with approximately a third of pre‐clinical pulmonary fibrosis cases manifesting progressive dyspnoea and worsening extent of abnormality on CT. Importantly, six cases died of respiratory failure during follow‐up.

Similar results have been reported by Hunninghake et al. with the prevalence of ILAs identified in the asymptomatic relatives of individuals with familial pulmonary fibrosis being extended to sporadic cases of pulmonary fibrosis [34]. Screening of asymptomatic relatives of individuals with sporadic pulmonary fibrosis identified ILAs in 14% and clinical ILD in 22%. The presence of an ILA was associated with abnormalities in lung function, *MUC5b* risk allele carriage, and telomere shortening. Similar findings to those above are expected in the Australasian population. The Australian IPF Registry reported that 13% of participants had a family history of pulmonary fibrosis [35]. A small study of first‐degree relatives of people with familial pulmonary fibrosis identified pre‐clinical ILD in seven of 15 cases (46.5%) [36].

Interestingly, despite the above findings, approximately half of surveyed ILD experts did not believe screening was indicated for individuals with a family history of pulmonary fibrosis, even when more than one family member had been affected [37]. This hesitancy is likely reflective of uncertainties about the optimal timing, impact, and risks of intervention in asymptomatic individuals. A survey of people with pulmonary fibrosis and their relatives demonstrated that many are concerned about the risk to unaffected family members [38]. An extension of the above‐mentioned study by Hunninghake et al. evaluated the psychological impacts in screened asymptomatic relatives [39]. Upon receiving a panel of results which included their telomere length, *MUC5B* status, telomere‐associated gene variants, gas transfer, and the presence or absence of an ILA, 42% of relatives reported mild or moderate regret, which, not surprisingly, was more common in those in whom the results were abnormal.

### 3.4 Connective Tissue Disease

Recent guidelines on the screening of individuals with systemic autoimmune rheumatic diseases (SARD) have conditionally recommended HRCT screening for those at increased risk of developing ILD [40]. It is now encouraged that individuals with systemic sclerosis, rheumatoid arthritis, idiopathic inflammatory myopathies, and mixed connective tissue disease with other ILD risk factors undergo HRCT at the time of diagnosis. While this is already happening in many cases through clinical convention, particularly in systemic sclerosis, the full impact of this recommendation is yet to be known, with a high potential for identification of mild/early changes. Interstitial changes may be present before the onset of rheumatological symptoms. Of 48 people with rheumatoid arthritis (RA) in one study, ILA was present in 62.5% [41]. Current and former smoking was associated with a higher prevalence of fibrosis. There was no association with exposure to methotrexate. Gochuico et al. identified pre‐clinical ILA in 33% of asymptomatic RA patients, many of whom progressed to RA‐ILD [42]. Chen et al. identified interstitial changes in 61% of patients with RA, the majority of whom were asymptomatic [43]. The presence of RA‐ILD was associated with older age, longer RA disease duration, higher articular disease activity, and the presence of physiological abnormalities. Similar findings of asymptomatic ILD have been reported for the other SARDs. Owing to the well‐established associations between ILD and many SARDs and the prognostic risk carried by the presence of ILD in SARD, it is suggested that in most cases, the discovery of an ILA in the context of a SARD be attributed to the rheumatological condition. One notable exception to this is the possibility of drug‐related pneumotoxicity, which may also occur in people with SARDs.

### 3.5 Occupational

There is limited data on ILAs in occupational settings. Harris et al. identified ILAs in 485 (32%) of 1513 participants of their Asbestos Review Program [44]. The program offers annual low‐dose CT to asbestos‐exposed individuals. In their predominantly older male cohort, time since first exposure, increasing tobacco exposure, and reported dyspnoea were associated with the presence of an ILA. Only a small fraction of those with an ILA demonstrated physiological deterioration during follow‐up. The detected ILAs frequently met criteria for asbestosis. Similar to interstitial changes detected in people with SARDs, the identification of interstitial changes in occupational cohorts must first be considered in terms of the contribution of the exposure to the identified interstitial changes. In many cases, the ILA may be better attributed to an early/mild pneumoconiosis rather than be defined along the lines of an ILA.

## 4 ILA and Long‐Term Health Outcomes

ILAs have consistently been associated with increased mortality across a number of studies [13, 21, 23, 45, 46]. A recent meta‐analysis comprising 88,325 participants, 7% of which had an ILA, reported an odds ratio for mortality associated with ILAs of 3.56 (95% CI, 2.19–5.81) [47]. Comprising data from the Framingham Heart Study, AGES‐Reykjavik Study, COPDGene study and ECLIPSE, Putman et al. identified a higher risk of death associated with ILAs in all cohorts after adjustment for covariates [13]. In the AGES‐Reykjavik cohort, those with an ILA had a higher rate of death of respiratory cause. Notably the mortality rate associated with ILAs was lower than that for clinical IPF. A subsequent analysis of the AGES‐Reykjavik cohort observed that risk of death was highest for those with ILAs meeting usual interstitial pneumonia (UIP) and probable UIP imaging patterns [48]. Interestingly the median survival for ILAs meeting UIP or probable UIP was not much longer than that associated with untreated IPF, and one might argue that such cases represent pre‐clinical IPF. Not surprisingly ILA progression was associated with mortality risk. Similar results were reported by Lee et al. in an Asian health screening population, with fibrotic ILAs associated with all‐cause mortality [45]. Not all deaths in those with ILA are respiratory related, and it is possible that the presence of an ILA may be a surrogate marker for other risk factors for death. An interesting association with ILAs is the observation of a higher incidence of acute respiratory distress syndrome and early mortality among critically ill patients [49].

Doyle et al. demonstrated that exercise capacity was reduced in those with an ILA in the COPDGene study, evidenced by shorter 6 min walk distances [50]. In an extension of this observation, Axelsson et al. observed lower grip strength, knee extension strength and thigh muscle mass in those with subpleural ILA [51]. Further data demonstrates that ILAs are associated with less functional independence and cases report worse self‐reported health [52]. It is not yet understood whether these observations represent a consequence of the ILA, or if these processes are reflective of underlying accelerated aging phenomena.

## 5 ILA Progression

Rates of ILA progression vary across cohorts (Table 2), with longer durations of follow‐up associated with a greater incidence of eventual ILA progression. In the AGES‐Reykjavik cohort, 73% of ILAs progressed over 5 years, with the greatest risk of progression observed in those with a definite fibrosis pattern, comprising architectural distortion with traction bronchiectasis and/or honeycombing [48]. Of the small number of cases meeting UIP or probable UIP radiologic patterns, all progressed. The odds of ILA progression increased when characterised by subpleural reticulation, lower zone predominance, and the presence of traction bronchiectasis. Additionally, the severity of traction bronchiectasis at baseline and its subsequent progression were predictive of mortality [53]. In comparison, the presence of centrilobular nodules significantly reduced the odds of progression.

Araki et al. characterised the decline in pulmonary function in participants of the Framingham Heart Study. Annual decline in FVC was 35 mL, 40 mL, and 64 mL for those without an ILA, non‐progressive ILA, and progressive ILA respectively [15]. This compares to an annual decline in FVC of approximately 240 mL in the placebo arms of the pivotal nintedanib phase 3 trials [54]. There is a suggestion that early IPF may demonstrate smaller declines in FVC than those reported in clinical trials [55]. Choi et al. utilised an automated, machine learning method to quantify ILA extent in two cohorts [56]. Each percent increase in ILA extent was associated with annual declines in FVC of 8.5–9.5 mL per year and 6‐min walk distance of 1.2 m.

## 6 Biomarkers in ILAs

### 6.1 *MUC5B* and Other Polymorphisms

The *MUC5B* promoter polymorphism (rs35705950) which has been associated with the risk of IPF, has consistently been associated with the presence of ILAs across a number of cohorts. Hunninghake et al. reported 2.8 times greater odds of ILA for each copy of the rs35705950 allele and 6.3 times greater odds of definite radiological fibrosis in the Framingham Heart Study [14]. Putman et al. identified similar associations with the *MUC5B* promoter SNP in participants of the AGES‐Reykjavik and COPDGene cohorts [16]. The minor allele frequency of the *MUC5B* promoter SNP was 12.7% in AGES‐Reykjavik, 10.3% in non‐Hispanic white participants from COPDGene, and 2% in African American participants from COPDGene. The *MUC5B* promoter was strongly associated with ILA (OR 2.1, 95% CI 1.8–2.4, *p* = 1 × 10^−26^) with a stronger association with subpleural ILA (OR 2.6, 95% CI 2.2–3.1, *p* = 1 × 10^−30^). There was no influence of the *MUC5B* promoter SNP on survival. Further analysis of the Framingham Heart Study cohort demonstrated that increasing copies of the *MUC5B* polymorphism were associated with ILA progression [15]. A relatively recent genome‐wide association study confirmed the *MUC5B* polymorphism association with ILAs but identified additional novel associations [57]. These included loci previously associated with IPF, including DPP9, DSP, FAM13A, and IVD, as well as associations near IPO11 and HTRE1, which have not been previously associated with IPF. How to incorporate genetic polymorphisms into clinical evaluation is yet to be defined.

### 6.2 Monocyte Count

Elevated monocyte counts have been associated with more aggressive IPF, and consequently, mortality across multiple IPF cohorts [58, 59, 60]. For ILA, a higher monocyte count was associated with the presence of ILA across the MESA, COPDgene, AGES‐Reykjavik, and ECLIPSE cohorts, as well as ILA progression in the AGES‐Reykjavik cohort [17]. Each standard deviation increment in blood monocyte count was associated with a 1.2–1.3 odds ratio for ILA. The association between monocyte count and the probability of ILA was linear in all four cohorts. Notably, smoking status, gender, the presence of emphysema, or age had no impact on the relationship of monocyte counts and the presence of ILA. In the AGES‐Reykjavik cohort, each standard deviation increment was associated with a 1.2 odds ratio for ILA progression at 5 years, independent of smoking and gender status. Monocyte activation studies were done in the MESA cohort, demonstrating an elevated proportion of activated monocytes in those with ILA.

### 6.3 Telomere Length

Telomere length and related gene variants have garnered increasing attention with regard to their association with IPF, in particular the familial form. Shorter telomere length has also been associated with the presence of ILAs. Putman et al. measured telomere length in COPDGene, AGES‐Reykjavik, and the Framingham Heart Study by a variety of methods [18]. In the COPDGene and AGES‐Reykjavik cohorts, there was a greater than twofold increase in the odds of ILA when comparing the shortest quartile of telomere length to the longest quartile. In the FHS, those with ILA had shorter telomeres than those without ILA. Similar associations between ILA and short telomere length have been identified in familial ILD screening cohorts [32, 34].

### 6.4 Proteome

Recently, the proteome of ILAs was evaluated in the AGES‐Reykjavik and COPDgene cohorts [61]. More than 4700 blood proteins were measured using DNA aptamers. OF almost 300 associations, the strongest associations with ILA were identified for SFTPB, SCGB3A1 (Secretoglobin family 3A member 1) and WFDC2 (WAP four‐disulfide core domain protein 2). Concentrations of SFTPB were linked with the presence of the MUC*5B* (mucin 5B) promoter polymorphism, and ILA progression was associated with levels of SFTPB and WFDC2. Notably, SFTPB has an association with the rs35705950 promoter polymorphism of *MUC5B*, the commonest risk factor for IPF [40] and the development of ILAs. This is a novel finding, and the hypothesis is that SFTPB is expressed in the distal lung zones with *MUC5B*. An imbalance of mucins versus surfactant protein may result in the development of IPF. Similar to other ILA biomarker associations, these proteins have a role in the pathogenesis of IPF. SFTPB is essential for the formation of surfactant protein and is found in low levels in plasma [62]. Raised levels of SFTPB are associated with a breach in the alveolar–capillary membrane [63]. Genetic variants for SFTPB and SFTPC are associated with the development of IPF. Raised WFDC2 (HE‐4) [human epididymis protein 4] has an unknown function and has been associated with IPF and expressed in epithelial cells and submucosal glands of the respiratory tract [64]. It is highly expressed in epithelial cells and submucosal glands in humans. SCGB3A1/UGRP2 [uteroglobin‐related protein 2]/HIN‐1 [high in normal 1] is a tumour‐suppressor gene secreted in the airways and has not been previously described in IPF. Molecules that have a negative association with ILA include WFIKKN2, an antagonist of GDF‐8 and GDF‐11 [61].

### 6.5 Automated CT/Radiomics

Automated, computer‐based methods to detect, diagnose, quantify, and prognosticate ILD are gaining increasing attention. The growing burden of ILA detection through lung cancer screening and widespread use of CT is likely to necessitate increasingly sophisticated computer‐based methods to ensure sensitivity and accurate prediction of ILAs that require further evaluation. A variety of methods have already been applied to ILA. Ash et al. developed a fully automated tool to detect ILA [65]. Using local histogram analysis, their tool had a sensitivity of 87.8% for ILA detection, which increased to 100% for fibrotic ILA. A subsequent study by Ash et al. applied a deep learning algorithm to ILA progression in the COPDGene Study [66]. Their data‐driven textural analysis (DTA) which has demonstrated robust data in people with IPF, was used to quantify ILA. DTA extent was correlated with FVC, 6‐min walk distance, and patient‐reported outcome measures. Using an established clinically relevant threshold for change in DTA, the authors demonstrated fibrotic progression in 17.6% of the cohort. Furthermore, progression of fibrosis was associated with mortality. They have demonstrated similar results in at‐risk individuals from a familial ILD screening cohort [67]. A number of other studies utilising various automated tools have demonstrated a similar ability to detect, quantify, and prognosticate ILA [56, 68, 69].

## 7 ILA vs. Preclinical ILD

The major opportunity afforded by the discovery of an ILA is the ability to institute early intervention in cases of ILD. The key step, therefore, is to differentiate early ILD from simple, inconsequential ILAs. Tomassetti et al. published recommendations on an approach to the incidental discovery of interstitial changes on CT [55]. They provide definitions that critically differentiate between ILA, early ILD, and mild ILD, enabling an ILD to be diagnosed in certain clinical circumstances. Pre‐clinical ILD may be differentiated from an ILA when identified in an individual at risk for ILD. This would apply to CT screening in individuals with an underlying SARD known to be associated with the potential for an ILD (e.g., rheumatoid arthritis). Additionally, a high index of suspicion should be given to a pre‐clinical ILD in asymptomatic family members of a person with pulmonary fibrosis who undergo CT imaging either for screening purposes or otherwise, in which interstitial changes are identified.

Subclinical ILD refers to the incidental discovery of an ILD in an individual without any specific elevated risk for an ILD. Tomasetti et al. suggest that pathological confirmation of an ILD is what differentiates this entity from an ILA [55]. Whether pathological confirmation is necessary in an incidentally discovered, asymptomatic individual is somewhat controversial. There remains an aversion to surgical lung biopsy given concern around the possibility of a mortality risk in people with IPF, and it is unclear whether newer techniques such as cryobiopsy and ENVISIA have sufficient sensitivity and specificity in such cases.

Rose et al. proposed the concept of ‘suspected ILD’ to apply to an ILA associated with any of the following features: radiological fibrosis, FVC less than 80% predicted and/or DLCO less than 70% predicted [70]. These criteria split the ILAs discovered in the COPDGene cohort in approximately half. Compared to individuals without an ILA or an ILA without features of ‘suspected ILD’, those meeting the criteria for ‘suspected ILD’ had worse survival.

Some lung cancer screening protocols have implemented standardised approaches to the follow‐up of ILAs. In the United Kingdom, ILAs detected through lung cancer screening with the following features generate a referral for further respiratory evaluation: an extent greater than 10%, 5%–10% with restrictive spirometry, a new ILA on a follow‐up scan, or a progressive ILA [71]. When cases meeting any of these criteria were subjected to ILD MDM evaluation, almost two thirds received an ILD diagnosis with management implications, with the other third remaining an ILA [71]. An extent of disease greater than 10% was present in almost all of the cases that received a distinct ILD diagnosis, suggesting that extensive ILAs are likely to be clinically classified as an ILD. Upperton et al. provided similar data demonstrating that clinical evaluation of selected lung cancer screen‐detected ILAs often yields an ILD diagnosis [25].

Studies that have tracked the outcome of ILAs have demonstrated that fibrotic ILA is associated with mortality risk. The presence of traction bronchiectasis and/or honeycombing is a strong risk factor for ILA progression and adverse survival. When ILAs were classified by UIP radiological criteria by Putman and colleagues [48], UIP and probable UIP patterns were associated with a striking mortality risk, with a median prognosis not much longer than that of clinical IPF. This data would suggest that when an ILA meeting UIP or probable UIP radiological criteria is discovered, particularly if extensive, consideration should be given to labelling that ILA as subclinical IPF (if no other cause is apparent) to enable early disease modifying therapy. When considering such a diagnostic label, it must be acknowledged that incidental/sub‐clinical IPF has not informed our existing literature and knowledge to a great extent. The prognosis of such patients is likely to be longer than clinically diagnosed IPF owing to the lead time effect. Additionally, tolerance of anti‐fibrotic side effects may be lower when the individual has no symptomatic awareness of the disease.

## 8 Clinical Approach to ILA

Radiological recognition of an ILA is the pre‐requisite for subsequent clinical evaluation. The benefit afforded by the discovery of an ILA is the ability to diagnose an ILD at the earliest possible timepoint, enabling the early commencement of disease‐modifying therapy. It is therefore critical that reporting radiologists incorporate an evaluation of the lung interstitium in any CT that captures lung fields of any extent (including the lung bases on abdominal CT). Oldham et al. have previously demonstrated that only 64% of ILAs detected through lung scanning screening were reported, with the finding of an ILA frequently omitted from the final radiological impression [72]. As a consequence, referral for further clinical evaluation was often missed. Standardisation of ILA reporting is therefore critical to their subsequent referral and clinical evaluation. Primary care clinicians will need instruction as to when to consider referral, given this is likely to be a new concept for many.

The first step in approaching an ILA is to determine whether the ILA is representative of a known ILD [1, 55]. The intention for all ILAs should be to differentiate those that represent an early ILD. The population from which the individual derives is the first step in making this distinction. Pre‐clinical ILD of known type should be considered in people with a SARD associated with the possibility of an ILD (e.g., rheumatoid arthritis, systemic sclerosis), following exclusion of other possible causes (e.g., drug toxicity, aspiration, pulmonary oedema). In this circumstance, the approach to management should follow that of the relevant clinical syndrome. Individuals with a family history of ILD should also be considered to have pre‐clinical familial ILD, with a low threshold to consider an IPF diagnosis to enable the commencement of anti‐fibrotic therapy, particularly when the ILD has fibrotic features radiologically. Outside of these population groups, an ILD diagnosis, even if unclassifiable, should be considered for ILAs that are extensive (greater than 10% lung involvement) or are associated with symptoms or physiological impairment.

Outside of the person originating from an at‐risk population, and with an extent of radiologic abnormality between 5%–10% in the absence of symptoms and/or physiologic impairment, a risk stratification approach should be adopted. This is most straightforward at the extremes of the risk spectrum. ILAs with the highest risk of progression are those meeting UIP radiologic criteria. When present, and in the absence of an explanation (e.g., suspicion of asbestosis), a diagnosis of subclinical IPF should be considered, and anti‐fibrotic therapy discussed with the patient [55]. ILAs that are not fibrotic nor basal –peripheral predominant are unlikely to progress and may be considered for discharge with risk factor modification (e.g., smoking cessation, minimisation of other environmental exposures) and safety netting [55].

The most challenging scenario in ILA management is the fibrotic ILAs and/or those with a basal‐peripheral predominance, occupying 5%–10% of the lung parenchyma, but without definite radiological features of UIP. Tomassetti et al. have suggested that bronchoalveolar lavage and biopsy should be considered on a case‐by‐case basis, and where UIP is confirmed, a diagnosis of subclinical IPF is made [55]. Where alternative to UIP histopathology is obtained, an alternative subclinical ILD diagnosis may be confirmed. In the absence of histopathology, the ILA is defined as at risk of progression, and as such, is subjected to ongoing follow‐up. However, the value of histopathology in the circumstance of incidental, asymptomatic, and physiologically preserved ILAs is unproven, and the yield and risks are undefined. Additionally, the studies on the histopathology of ILAs discussed earlier in this document demonstrate that ILAs might demonstrate UIP pathology, but that in itself does not necessarily predict an IPF disease trajectory, although the risk is certainly heightened. Cryobiopsy as an alternative to surgical lung biopsy may not be sufficiently sensitive, owing to the mild, subpleural involvement of the abnormality, although this is also unknown. Whether biopsy represents a solution to this intermediate risk ILA group requires further evaluation. It is likely that more refined risk stratification tools, including various‐omic modalities, will prove more effective for risk assessment.

**FIGURE 4:**
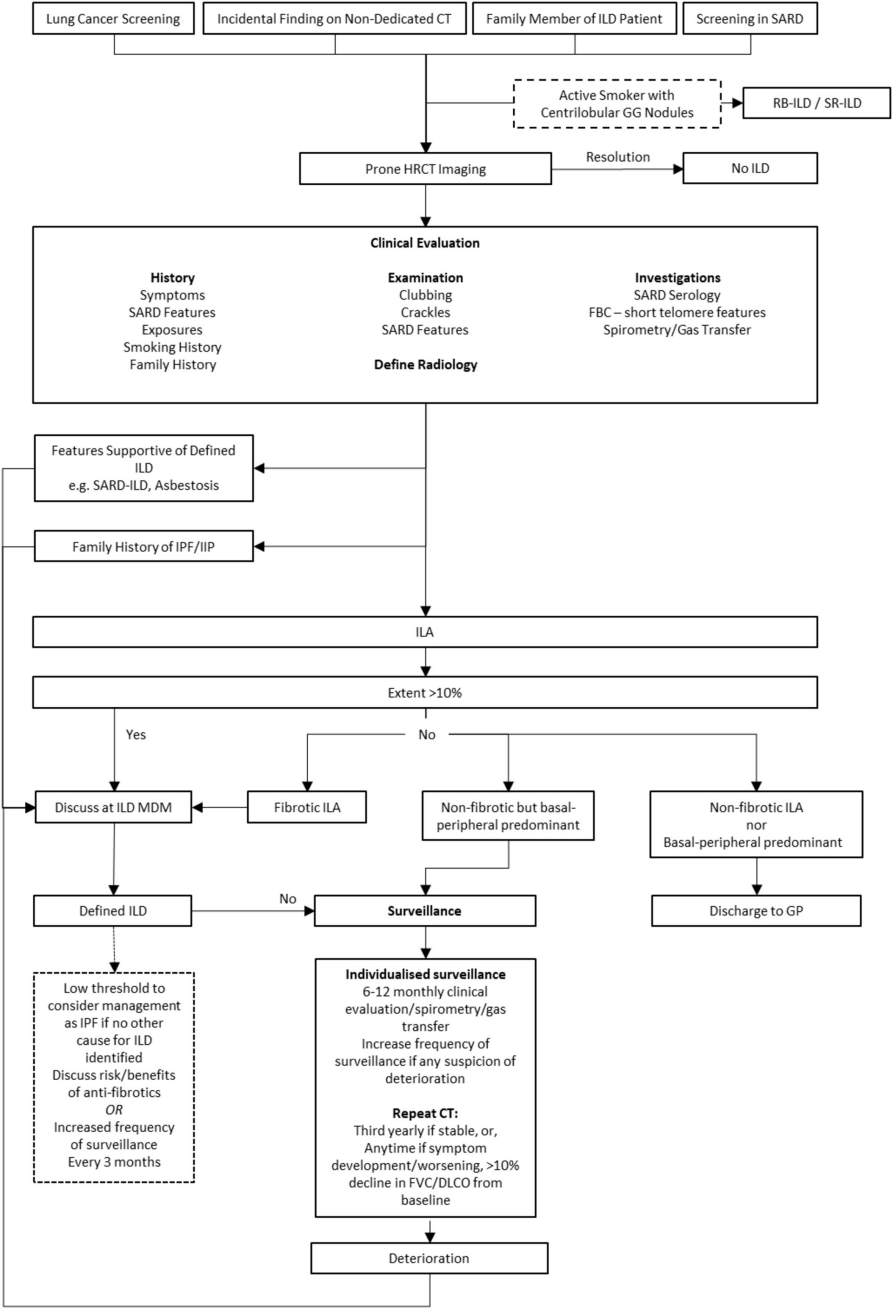
Proposed algorithm for the approach to interstitial lung abnormalities.

For the remaining ILAs at risk of progression, the optimal interval and duration of surveillance remain to be defined. While ILA progression is associated with FVC decline, the magnitude of annual FVC decline in progressive ILAs is relatively small [56]. Tomassetti et al. graphed the annual FVC decline across various interstitial entities [55]. Annual FVC decline in the absence of an ILA was listed as −35 mL, for a non‐progressive ILA −40 mL, and for a progressive ILA −64 mL; in comparison to −83 mL for ‘real‐life’ early IPF. Such small differences are unlikely to be clinically distinguishable. When to repeat HRCT imaging outside of routinely scheduled scanning as part of lung cancer screening is also undefined. Population cohort studies demonstrate that ILA progression becomes more prevalent with longer re‐scanning intervals. Park et al. performed a retrospective evaluation of ILA follow‐up to determine the optimal follow‐up strategy [73]. Based on a median time to ILA progression of 3.2 years, they suggest CT re‐evaluation at 3‐year intervals. When to cease surveillance remains to be defined and will be critical to managing the follow‐up burden of ILA surveillance. In Figure 4, we propose a cautious approach to at‐risk ILA surveillance; however, this approach should be modified on a case‐by‐case basis.

The final clinical consideration with regard to the identification of an ILA is its potential impact on surgery or systemic cancer therapies. Associations between ILAs and adverse outcomes have been identified with cancer surgery [74, 75], radiotherapy [76, 77], chemotherapy, and newer targeted‐ and immune‐therapies [78, 79, 80]. As such, recognition of an ILA on pre‐treatment CT scans is fundamental to the evaluation of risk and planning of appropriate treatment. Identification of ILAs that represent subclinical ILD may enable commencement of therapies that modulate this risk. There is some data to suggest that the risk of acute exacerbations and mortality is less in patients with IPF undergoing lung cancer surgery while receiving anti‐fibrotic therapy [81, 82, 83]. Whether this benefit extends to systemic therapies is unknown. In patients with an ILA undergoing surgery requiring mechanical ventilation, it has been recommended that a low‐volume, low‐pressure ventilatory approach be adopted [1].

## 9 Future Directions

The evolving understanding of ILA risk factors and biomarkers suggests potential opportunities for targeted screening programmes. High‐risk populations, particularly those with a family history of pulmonary fibrosis and individuals newly diagnosed with SARD, may benefit from systematic screening approaches. Recent advances in genetic testing, including *MUC5B* promoter polymorphism status and telomere length assessment, combined with emerging blood‐based biomarkers and proteomics, could enable risk stratification to identify individuals most likely to benefit from CT screening. However, implementation of such screening programmes will require careful consideration of cost‐effectiveness, radiation exposure, and the psychological impact of early diagnosis in the context of uncertain therapeutic implications.

## 10 Conclusion

ILAs represent a new and growing challenge for respiratory clinicians. With the implementation of lung cancer screening, clinicians will be faced with an influx of ILAs necessitating risk stratification within an already burdened health care system. The identification of risk factors such as older age, smoking history, and genetic polymorphisms, along with emerging biomarkers, offers opportunities for risk stratification and targeted surveillance. The clinical approach to ILAs requires a delicate balance between avoiding unnecessary intervention for ‘benign’ ILAs and ensuring timely recognition and treatment of ILAs that represent early, preclinical ILD. Risk stratification based on radiological features, extent of involvement, and associated clinical and physiological findings provides a framework for management decisions, ranging from conservative surveillance to consideration of anti‐fibrotic therapy in high‐risk cases. As our understanding of ILAs evolves, several key areas warrant further investigation. These include refining risk prediction models, evaluating the impact of early intervention on disease progression, and exploring the potential role of genetics and biomarkers in risk assessment.

## Author Contributions

**Yuben Moodley:** conceptualization (equal), writing – original draft (equal), writing – review and editing (equal). **John A. Mackintosh:** conceptualization (equal), writing – original draft (equal), writing – review and editing (equal).

## Conflicts of Interest

Y.M. is an Editorial Board member of Respirology and a co‐author of this article. He was excluded from all editorial decision‐making related to the acceptance of this article for publication. J.A.M. declares honoraria from Boehringer Ingelheim, unrelated to the manuscript.
